# Resilience of patients with skin diseases in France: A cross‐sectional study

**DOI:** 10.1111/jdv.18681

**Published:** 2022-10-31

**Authors:** Bruno Halioua, Christine Patras de Campaigno, Stéphanie Merhand, Benedicte Charles, Marie France Bru, Clemence Legrand, Christopher Beausillon, Helene Raynal, Charles Taieb, Marie‐Aleth Richard

**Affiliations:** ^1^ Private Dermatologist Dermatologist Paris France; ^2^ Association Française Prurigo Nodulaire Agen France; ^3^ Association Française de l'Eczéma Redon France; ^4^ France Psoriasis Paris France; ^5^ Association Française pour la Recherche sur l'Hidrosadénite St Benoit France; ^6^ France Acné Adolescents Adultes Vincennes France; ^7^ Solidarité Verneuil Villeurbanne France; ^8^ European Market Maintenance Assessment Fontenay sous‐Bois France; ^9^ Dermatology Department, CEReSS‐EA 3279, Research Centre in Health Services and Quality of Life Aix Marseille University University Hospital Timone Marseille France

Dear Editor,

An individual's resilience defines his or her ability, in the face of a stressful environment or adversity (an event, an illness) to maintain a good quality of life.^1^ Resilience represents the quality that allows one to adopt a positive attitude and good adjustments and to sustain relatively stable well‐being despite adverse life events. Resilience has been studied in different populations, age groups and chronic conditions, but little is known about this issue in patients with skin diseases (SD).^2^

This study aimed to assess the level of resilience and related factors among patients with SD. The validated French version of the Connor–Davidson Resilience Scale1 (CD‐RISC)^2^, ^3^, ^4^ that was used is a 25‐item generic resilience instrument with three subscales: tenacity, strength and optimism. Items were scored on a 5‐point Likert scale ranging from 0 (never) to 4 (very often). A total score is calculated as the sum of all questions and ranges from 0 to 100. A higher score is considered to express better resilience.^2^, ^3^ There is no published cut‐off score.

We conducted [Between February and March 2022], a population‐based study on representative and extrapolable samples of the French general population aged 18 years or more according to the quota method (age, gender, geographical location and level of income). Participants we selected using a stratified, proportional sampling with replacement design. Data were collected using a web‐based online survey. All participants were asked to fill in the questionnaire included the CD‐RISC.

The entire sample was asked about the presence or absence of a skin disease. The presence of a cardiovascular [high impact in terms of mortality], osteoarticular [high impact in terms of mobility/autonomy] or other chronic diseases was also sought.

Student's *t* test and Pearson's *r*‐ratio were used. All results are expressed as the mean ± SD. Comparisons between groups were performed using a two‐sample Student's *t* test; Pearson's correlation coefficients were used to examine bivariate correlations. A *p* < 0.05 was considered to represent a statistically significant difference.

Of the 3652 individuals reporting a SD [Figure 1], 1069 were identified as having exclusively SD: psoriasis (*n* = 255, 23.9%); acne (*n* = 263, 24.6%), atopic dermatitis (*n* = 301, 28.2%), hand chronic eczema (*n* = 49, 4.6%), rosacea (*n* = 92, 8.6%), urticaria (*n* = 46, 4.3%), vitiligo (*n* = 46, 4.3%) and hidrosadenitis suppurativa (*n* = 17, 1.6%). A total of 39.4% were men, and 60.6% were women. Participants' ages ranged from 18 to 82 (41.01 ± 15.94) years. In addition, we identified 1128 patients with exclusive osteoarticular diseases, 394 with exclusive cardiovascular diseases and 6878 individuals without any chronic disease. These are considered healthy controls.

**FIGURE 1:**
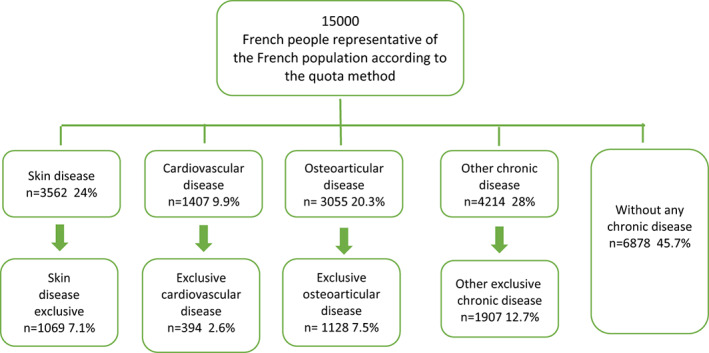
Populations flowchart

The mean score for resilience of patients with SD was 61.2 (SD ± 14.4). Resilience Scale total scores were significantly lower in patients with SD than in healthy individuals (61.2 vs 62.5 *p* = 0.007) and particularly for confidence in one's intuition and tolerance to stress (16.6 vs 17.1, 0.0001), positive acceptance of change (12.7 vs 13, *p*: 0.007), perceived control (7.2 vs 7.5, *p* 0.006) but spirit tenacity (20.5 vs 20.7, *p* 0.15) and spirituality (4.1 vs 4.1; *p* 0.54). Scores for resilience were significantly lower in patients with SD than in patients with osteoarticular diseases (61.2 vs 62, 9 *p* = 0.004). There was no statistically significant difference in resilience levels with cardiovascular diseases (61.2 vs 60.10, *p* 0.18).

Among patients with SD, significantly lower resilience was reported in patients who lived in rural areas compared to those who lived in urban areas (58.7 vs 61.5 *p* 0.03) who were not married compared with marred (60.1 vs 61.9, *p* 005) and with an annual income (≤24 k€: 59.1 vs >24 k€: 62.4, *p* 0.0004) Table 1. No statistically significant difference was found in resilience levels relative to other sociodemographic factors (age, gender and professional status) Table 1.

**TABLE 1:** CD‐RISC score by population typology

	Numbers (%)	CD‐RISC (mean ± SD)
Global population	15,000 (100%)	61.6 ± 14.3
Healthy controls	6878 (45.7%)	62.5 ± 14.4
Exclusive skin disease	1069 (7.1%)	61.2 ± 14.4
Exclusive cardiovascular diseases	394 (2.5%)	60.1 ± 14.3
Exclusive osteoarticular diseases	1128 (7.5%)	62.9 ± 13.0
More than 2 skin diseases (without any other chronic disease)	148 (1%)	57.3 ± 17.3

Moreover, we observe that the number of skin diseases has an impact on the resilience of individuals in fact the patients with more than 2 skin diseases had a significantly lower resilience score compared with individuals with one skin disease (57.3 vs 61.2, *p* 0.01).

To the best of our knowledge, this is the first study assessing resilience in a large group of patients with SD. We have demonstrated that the level of resilience with skin disease's patients was not significantly different to the level of resilience in patients with cardiovascular diseases. Understanding the factors that affect a higher level of resilience can have important clinical implications and can represent a guiding principle for designing psychological interventions that would accelerate recovery and improve the quality of life of dermatological patients. It would probably be necessary and appropriate to identify patients with low resilience and to refer them to appropriate psychological care if possible or to take a minimum of time to talk about it during the consultation. Further research is required to better understand how resilience influences patient engagement with dermatological services, adherence to treatment, attitude towards healthy living and clinical outcome of patients over time.

## FUNDING INFORMATION

This study was granted by the “Printemps des Maladies Chroniques Inflammatoires de la Peau” with institutional support from Almirall, Leo‐Pharma, Pfizer, Bioderma, Pierre Fabre, Amgen, Sanofi, La Roche Posay, Cerave, Vichy and UCB pharma.

## CONFLICT OF INTEREST

None declared.

## Data Availability

The data that support the findings of this study are available from the corresponding author upon reasonable request.

## References

[1] Herrman H , Stewart DE , Diaz‐Granados N , Berger EL , Jackson B , Yuen T . What is resilience? Can J Psychiatry. 2011;56(5):258–65. 10.1177/070674371105600504 21586191

[2] Kim GM , Lim JY , Kim EJ , Park SM . Resilience of patients with chronic diseases: A systematic review. Health Soc Care Community. 2019;27(4):797–807. 10.1111/hsc.12620 30027595

[3] Connor KM , Davidson JR . Development of a new resilience scale: The Connor‐Davidson Resilience Scale (CD‐RISC). Depress Anxiety. 2003;18(2):76–82. 10.1002/da.10113 12964174

[4] Guihard G , Deumier L , Alliot‐Licht B , Bouton‐Kelly L , Michaut C , Quilliot F . Psychometric validation of the French version of the Connor‐Davidson Resilience Scale. Encephale. 2018;44(1):40–5. 10.1016/j.encep.2017.06.002 28870690

